# A New Approach to Atopic Dermatitis Control with Low-Concentration Propolis-Loaded Cold Cream

**DOI:** 10.3390/pharmaceutics13091346

**Published:** 2021-08-27

**Authors:** Bianca Aparecida Martin, Camila Nunes Lemos, Luciana Facco Dalmolin, Caroline Arruda, Íris Sperchi Camilo Brait, Maurílio de Souza Cazarim, Estael Luzia Coelho da Cruz-Cazarim, Paula Carolina Pires Bueno, Maurílio Polizello Júnior, Leonardo Régis Leira Pereira, Renata Nahas Cardili, Renata Fonseca Vianna Lopez

**Affiliations:** 1 School of Pharmaceutical Sciences of Ribeirão Preto, University of São Paulo, Ribeirão Preto 14040-903, SP, Brazil; bianca.apmartin@usp.br (B.A.M.); camila.lemos@usp.br (C.N.L.); lucianafd@usp.br (L.F.D.); carolinearruda@alumni.usp.br (C.A.); iris.camilo@alumni.usp.br (Í.S.C.B.); maurilio.cazarim@farmacia.ufjf.br (M.d.S.C.); estaelcruz@fcfrp.usp.br (E.L.C.d.C.-C.); bueno@mpimpgolm.mpg.de (P.C.P.B.); polinet@fcfrp.usp.br (M.P.J.); lpereira@fcfrp.usp.br (L.R.L.P.); 2 Ribeirão Preto Medical School, University of São Paulo, Ribeirão Preto 14040-903, SP, Brazil; nahas-renata@uol.com.br

**Keywords:** raw beeswax, atopic dermatitis, green propolis, anti-inflammatory property

## Abstract

Atopic dermatitis (AD) is a chronic inflammatory skin disease that is difficult to treat. Traditional cold cream, a water-in-oil emulsion made from beeswax, is used to alleviate AD symptoms in clinical practice, although its effectiveness has not been scientifically proven. The addition of propolis has the potential to impart anti-inflammatory properties to cold cream. However, in high concentrations, propolis can trigger allergic reactions. Thus, the objective of this work was to develop a cold cream formulation based on purified beeswax containing the same amount of green propolis present in raw beeswax. The impact of adding this low propolis concentration to cold cream on AD control was evaluated in patients compared to cold cream without added propolis (CBlank). Raw beeswax was chemically characterized to define the propolis concentration added to the propolis-loaded cold cream (CPropolis). The creams were characterized as to their physicochemical, mechanical, and rheological characteristics. The effect of CPropolis and CBlank on the quality of life, disease severity, and skin hydration of patients with AD was evaluated in a triple-blind randomized preclinical study. Concentrations of 34 to 120 ng/mL of green propolis extract reduced TNF-α levels in LPS-stimulated macrophage culture. The addition of propolis to cold cream did not change the cream’s rheological, mechanical, or bioadhesive properties. The preclinical study suggested that both creams improved the patient’s quality of life. Furthermore, the use of CPropolis decreased the disease severity compared to CBlank.

## 1. Introduction

Atopic dermatitis (AD), or atopic eczema, is a chronic inflammatory disease of the skin associated with atopy with outbreak evolutions [1]. The most common symptoms associated with the disease are severe itching in specific skin sites and eczematous lesions with cyclic evolution [2]. In the acute phase of the disease, the lesions appear as poorly defined edemas and erythemas, while in the chronic phase, they become more defined, lichenified, desquamative, and located mainly in regions of body folds. In addition, patients with the disease have severe xerosis [3,4].

The prevalence of atopic dermatitis is significantly increasing in the world. Approximately 10–20% of adults with AD reported severe disease. In 2010, almost 230 million people were diagnosed with eczemas [5].

The pathophysiology of AD involves multiple interrelated etiologies, such as genetic, environmental, and immunological factors, which affect the barrier function of the skin, primarily conferred by the stratum corneum, and trigger inflammatory responses [4,6].

Thus, the stratum corneum of the atopic patient undergoes changes in lipid chains and water content, causing xerosis and increased pH. It is supposed that these changes are caused mainly by the deficiency of filaggrin, an essential protein involved in the formation of the stratum corneum, and by the organization of the lipid matrix, with ceramides with chain size and distinct proportions of healthy skin [3].

The altered stratum corneum of the AD patient leads to an increase in transepidermal water loss and consequent skin dehydration [7]. The dendritic terminals of Langerhans cells, present in the viable epidermis, are also more exposed to antigens that generally would not be able to cross the stratum corneum, triggering exacerbated inflammatory and immune responses [4]. The inflammatory response culminates in the production of IgE antibodies and the release of several interleukins (IL), tumor necrosis factor-alpha (TNF-α), and interferon-gamma (IFN-γ) [4,8,9,10]. The AD patient’s skin also lacks antimicrobial peptides responsible for defending the body against the invasion of bacteria, viruses, and fungi. Consequently, patients with AD are more likely to develop microbial infections [4].

Topical administration of creams is the main form of treatment for AD and can reduce the signs and symptoms of the disease [4]. However, the creams usually contain corticosteroids or calcineurin inhibitors, the prolonged use of which causes severe adverse effects, such as stretch marks, skin atrophy, and hypopigmentation [4,11], making the practice unsafe [12].

Therefore, considering the chronicity character of AD, therapeutic alternatives are needed to delay the appearance of lesions and avoid crisis periods [13]. Topical application of non-drug-added creams, known as base creams, is used to avoid the constant use of corticosteroids and calcineurin inhibitors. Base creams developed with suitable emollients are considered a safe therapeutic approach to skin protection [14]. Their use can also increase the hydration of the stratum corneum and restore the skin’s barrier function [14], which are valuable properties for AD treatment.

Cold cream is one of the oldest dermatological pharmaceutical dosage forms known, developed in the 2nd century by Galen. It is a water-in-oil semisolid emulsion; its original formulation consisted primarily of beeswax, mineral oil, and water. Because the cold cream has a high lipid content, it is used in situations in which high emollience is required, such as psoriasis, AD, and skin disorders characterized by intense skin dryness [15,16,17].

The effectiveness of cold cream uses in the treatment of AD patients has been observed in a clinic [18], but with few studies reported in the scientific literature [19].

In recent years, pharmacists from the Teaching Pharmacy of the Faculty of Pharmaceutical Sciences of Ribeirão Preto, University of São Paulo (USP), Brazil, have manipulated some batches of cold cream made with about 13% raw beeswax, rather than purified beeswax. Patients with AD who used these creams informally reported improvement in the signs and symptoms of the disease.

Considering the origin of beeswax, we hypothesized that the observed improvement would be related to propolis remnants in raw beeswax. Propolis is a natural resinous substance formed by several complex components collected by bees from different plant species and plant parts (branches, flowers, pollen, and tree exudates). The collected resin is deposited in the hive with saliva and insect enzymes to seal the cracks, maintaining temperature and preventing the proliferation of microorganisms [20,21].

Propolis has several properties that may benefit AD control, including anti-inflammatory, antifungal, antibacterial, antioxidant, immunomodulatory, antiulcerogenic, and antimutagenic [22,23]. Its immunomodulatory, anti-inflammatory, and antioxidant properties are attributed mainly to the phenolic compounds present in its composition [24,25,26,27].

In the present study, we developed purified beeswax-based cold cream formulations loaded or not with propolis extract. The propolis-loaded formulation was standardized with the same amount of green propolis present in about 13% raw beeswax. Therefore, raw beeswax was chemically characterized to verify the presence of propolis chemical markers. We investigated the influence of green propolis on AD control and the anti-inflammatory properties of low concentrations of propolis. The impact of cold cream loaded or not with propolis on quality of life, disease severity, and skin hydration of patients with AD was evaluated.

## 2. Materials and Methods

### 2.1. Material

Purified beeswax (lot: 1321) was purchased from GM Comércio de Ceras e Derivados, Socorro, Brazil; raw beeswax (lot: 153A) was obtained from Vale do Mel, Ribeirão Preto, Brazil; and dry green propolis was purchased from Apis Flora, Ribeirão Preto, Brazil. Ethanol was purchased from Labsynth, Diadema, Brazil; and formic acid, dimethylsulfoxide (DMSO), and sodium borate were purchased from Synth, Diadema, Brazil. Isopropanol, hexane, and dichloromethane were purchased from Honeywell, Charlotte, NC, USA; and acetonitrile and methanol from JT Baker, Mexico City, Mexico. Pyridine was purchased from Vetec Quimica, Duque de Caxias, Brazil. N,O-Bis(trimethylsilyl)trifluoroacetamide (BSTFA), trimethylchlorosilane (TMCS), veratraldehyde, *E. coli* liposaccharide (LPS), and propidium iodide were all purchased from Sigma Aldrich, St. Louis, MO, USA. Antibiotic-antimycotic solution (100×), fetal bovine serum (FBS), and Dulbecco’s modified Eagle’s medium (DMEM) were obtained from Gibco, Grand Island, NY, USA. Liquid petroleum jelly was purchased from Fagron, São Paulo, Brazil, and Glydant Plus Liquid^®^ was obtained from LSI, Basel, Switzerland. Solid petroleum jelly, cetostearyl alcohol, ethoxylated cetostearyl alcohol 20, cocoa butter, glyceryl monostearate, and butylhydroxytoluene were obtained from Mapric, São Paulo, Brazil. Pig ear skin was purchased from Frigorífico Olhos D’água, Ipuã, Brazil. Deionized water (18.2 MΩ-cm at 25 °C) (Milli-Q, Direct-Q 3 UV, Millipore, Bedford, MA, USA) was used to prepare all solutions.

### 2.2. Composition of Beeswax

The fatty components present in the purified and raw beeswax, obtained from wild bees and *Apis mellifera*, respectively, were identified with a gas chromatograph coupled to a mass spectrometer (CG-MS) (Shimadzu, QP-2010, Kyoto, Japan) [28]. Briefly, 1 g of beeswax was solubilized in 10 mL of hexane. This solution was added of 10 mL of methanol/water (9:1), followed by agitation and separation of the phases, resulting in a hexane fraction and a methanolic fraction. Then, 10 mL of dichloromethane was added to the hexane fraction, which, after separation, gave rise to the hexane fraction and the CH_2_Cl_2_-Hex fraction. The methanolic fraction was added of 5 mL of dichloromethane/hexane (1:1), which, after separation, gave rise to the dichloromethane/hexane fraction and the second methanolic fraction. This resulting methanolic fraction was mixed with 5 mL more dichloromethane, giving rise to the CH_2_Cl_2_-MeOH fraction and the final methanolic fraction after phase separation. Each of the five fractions obtained (hexane, CH_2_Cl_2_-Hex, dichloromethane/hexane, CH_2_Cl_2_-MeOH, and final methanolic) was evaporated and added to 550 μL of BSTFA and TMCS solution (98:2) as silylation agents, in addition to 100 μL of pyridine. This mixture was kept in a water bath at 80 °C for 30 min. The injections were performed at 260 °C, and the separation of the compounds was performed using a DB-5MS column measuring 30 m × 0.25 mm × 0.25 μm (Agilent Technologies, Santa Clara, CA, USA), maintained at 80 °C. The helium pressure was 187.1 kPa at a linear velocity of 31.9 cm/s, with column flow of 1.5 mL/min. The mass spectrum was recorded in SCAN mode at 70 eV, with an ion source temperature of 250 °C. The mass range was scanned in full scan mode from 40 m/z to 700 *m*/*z* every 0.3 s. Based on the chromatograms obtained, the mass spectrum corresponding to each peak was compared with the theoretical spectra of the NIST11, NIST11-S, WILEY7, NIST08, and FFNSC1.3 databases, using the similarity index.

### 2.3. Chromatographic Profiling of Raw Beeswax and Quantitative Analysis of Artepillin C

One gram of raw beeswax was solubilized in a water bath in 10 mL of hexane, and this solution was partitioned with 10 mL of methanol and water (9:1) three times. The methanolic fractions were combined, filtered with 0.45 μm filters, and evaporated. The resulting precipitate was solubilized in 1 mL of methanol and injected into the HPLC (Waters, Milford, MA, USA) equipped with a binary pump, automatic injector, diode array detector, and software (Empower, MA, USA). The column used was a C18 (Phenomenex LUNA, Torrance, CA, USA) of 250 mm × 4.6 × 5 μm with a precolumn C18 of 4 mm × 3.0 mm. The mobile phase was composed of (A) purified water containing 0.4% formic acid, 5% methanol, and 2% isopropanol, and (B) 2% isopropanol in acetonitrile, with a flow of 1 mL/min in a concentration gradient, using 20% B for 3 min, 20–25% B for 3–4 min, 25% B for 4–15 min, 25–45% B for 15–20 min, 45% B for 20–40 min, 45–60% B for 40–45 min, 60–80% B for 45–68.83 min, 80–20% B for 68.86–70 min, and 20% B for 70–80 min. The injection volume was 15 μL, the column oven temperature was 30 °C, and the quantification wavelength was 300 nm [29].

The chromatographic profile of the beeswax extract was compared with the chromatographic profile of a standardized green propolis extract [30] eluted under the same chromatographic conditions.

For quantitative analysis, 3,5-diisopentenyl-4-hydroxycinnamic acid (artepillin C) was used as a marker of green propolis. A calibration curve was constructed in the range of 0.38 to 112 μg/mL of artepillin C in methanol. Each point of the curve contained 25 μg/mL of veratraldehyde as an internal standard [29].

### 2.4. Preparation of Standardized Green Propolis Extract

We mixed 50 g of dry green propolis with 500 mL of ethanol and purified water (7:3). This mixture was macerated (Marshall Scientific, Innova 4300, Hampton, NH, USA) at 120 rpm, 35 °C, for 24 h and filtered. This process was repeated three times, and the filtrate was evaporated in a rotary evaporator (Rotaevaporator, R-210, Vacuum Controller, V-855, Vacuum Pump, V-700, Heating Bath, B-491, Buchi, Switzerland) to obtain approximately 44 g of green propolis extract. The artepillin C content in the dry extract was determined using the analytical curve and chromatographic method described above. The concentration of artepillin C determined was 117.36 ± 2.74 mg/g of dry extract.

### 2.5. Anti-Inflammatory Activity of Green Propolis Extract

AMJ-2 macrophages were cultured in DMEM supplemented with 10% heat-inactivated FBS and 1% (*v*/*v*) antibiotic–antimycotic solution. The cells were plated in 24-well plates (Kasvi, China) at a 4 × 10^5^ cells/well density. The cells were added to 34, 60, or 120 ng/mL of standardized green propolis extract dissolved in a complete DMEM culture medium containing 0.05% DMSO, corresponding to 4–14 ng/mL of artepillin C, in addition to 10 μL of an LPS solution at 100 μg/mL. The cells were incubated at 37 °C and 5% CO_2_ for 24 h [31]. After this period, 50 μL of the undiluted supernatant was collected, and the concentrations of IL-6, TNF-α, IL-17, and INF-γ were analyzed by Elisa using specific kits, following the manufacturer’s specifications (R&D Systems Corporation, Minneapolis, MN, USA). The analysis was performed at 450 nm in a SpectraMax Paradigm microplate reader (SoftMax Pro 6.2.1, Molecular Devices, San Jose, CA, USA) [32].

The cytotoxicity of the green propolis extract at studied concentrations was evaluated by flow cytometry (BD FACSCanto II, BD Biosciences, San Jose, CA, USA). Nonviable cells were stained with propidium iodide and evaluated at 570–700 nm. The experiments were carried out in three replicates.

### 2.6. Preparation and Characterization of Cold Creams

Two creams were prepared: one without propolis (CBlank) and the other containing green propolis extract (CPropolis). The creams were prepared by heating separately, at 70 °C, the aqueous phase, composed of 1% sodium borate and purified water qsp 100; and the oily phase, composed of 13.5% purified beeswax, 1.5% cetostearyl alcohol, 0.3% cetostearyl alcohol ethoxylate 20, 0.05% BHT, 1.5% cocoa butter, 1% monostearate of glyceryl, 13.5% solid petroleum jelly, and 32% liquid petroleum jelly. The aqueous phase was poured into the oil under mechanical stirring (Fisatom, 722, São Paulo, Brazil) at 245 rpm for 5 min and maintained at 300 rpm until cooled (25 ± 5 °C). Then, 0.2% of the preservative (Glydant Plus Liquid^®^, Basel, Switzerland) was added and homogenized.

A total of 2.3 mg of standardized green propolis extract, corresponding to 2.7 μg/g of artepillin C, was added to 100 g of CPropolis by spatulation. This amount of artepillin C roughly matched that found in 13.5% raw beeswax.

The creams were characterized for droplet size, as well as mechanical and bioadhesive properties.

#### 2.6.1. Diameter of Droplets

The droplet diameter was evaluated, in duplicate, by optical microscopy (Nikon, Eclipse E100, New York, NY, USA). The cream samples were diluted in liquid petroleum jelly, the droplets counted in a Neubauer chamber, and the average number of droplets per unit weight of cream (*N*) was determined according to Equation (1):

(1) $$N=\frac{ñE}{VQ\rho_{d}}$$

where *ñ* = average number of droplets per quadrant, *Q* = quantity in g of the internal phase of the emulsion per g of emulsion, *E* = dilution, ρd = dilution liquid density (0.88 g/mL), and *V* = volume under the observation square (25 · 10^−8^ cm^3^). From the *N* value obtained and the density of the internal phase (ρ_i_), which was basically composed of water (1 g/cm^3^), the volume-number diameter (*d_VN_*) of the droplets (Equation (2)) was calculated:

(2) $$d_{VN}=\sqrt[{3}]{\frac{6}{\rho_{i}N_{w}\pi }}\;$$

#### 2.6.2. Mechanical Properties

A texture profile analysis (TPA) of the formulations was carried out with a TA-XT texture analyzer (Stable Micro Systems Ltd., Surrey, UK) to determine the mechanical properties. Cream samples measuring 10 g were placed in a beaker and left at room temperature (25 °C) for 24 h before the test, avoiding the introduction of air bubbles. The analytical polycarbonate probe (10 mm diameter) was compressed twice in the interiors of the samples, with a test speed of 2 mm/s^−1^, a depth of 5 mm, and a delay period of 15 s between the first and second compressions [33]. The analyses were performed 10 times for each sample at 25 °C. Based on force vs. time graphs, hardness (force required to attain a given deformation), compressibility (work required to deform the product during the first compression of the probe), cohesiveness (ability to restructure the formulation after removing from the vial), and elasticity (deformation rate after applying a force) of the samples were calculated using the software Exponent (Stable Micro Systems Ltd., Surrey, UK).

#### 2.6.3. Bioadhesive Properties

The force required to detach the cream from the surface of a skin sample was evaluated using the texture analyzer in tension mode [34]. The skins used were obtained from pig’s ears collected immediately after the slaughter of the animals. Briefly, the ears were kept at approximately 4 °C while transported to the laboratory, where the skins were dissected. The skin samples were placed on the inferior accessory of the equipment, and the cream was fixed on the top probe (bioadhesion ring). The probe was lowered until it entered into contact with the skin’s surface, maintaining compression of 0.5 N for 60 s. After this period, the probe was raised at a 1 mm/min^−1^ speed until the cream detached from the skin. The force necessary to detach the cream from the skin (detachment force) and the work of bioadhesion (area under the force vs. distance curve) were determined using the software Exponent (Stable Micro Systems, Surrey, UK). The analyses were performed four times for each sample.

### 2.7. Stability

The creams were stored at room temperature (25 ± 2 °C) in wide-mouthed white polyethylene jars with a screw cap for 60 days (time and condition in which they were under the care of patients in the clinical trial). After 1, 15, 30, 45, and 60 days, samples were analyzed for organoleptic characteristics and rheological behavior.

The rheological properties were obtained in a rheometer (TA, Discovery HR-2 Hybrid Rheometer, New Castle, DE, USA) with controlled shear rate, in flow mode, and employing cone-plate geometry (40 mm diameter) with a 59 μm gap between the cone and plate, and a 2° angle. The analyses were performed three times for each sample at 25 °C. Approximately 3 g of the creams were applied on the rheometer plate and kept at rest for 60 s, ensuring the minimum shear. The flow curves (ascending and descending) were obtained with increasing shear rate from 1 to 100 s^−1^ over 30 s. Hysteresis areas were calculated using the Origin 6.1 software (Northampton, MA, USA). The ascending and descent curve integrals were obtained, and the ascendant values were subtracted from the descending ones.

### 2.8. Statistical Analysis

Comparisons of the means of two populations were performed using the *t*-test. In the case of three or more populations, the data were evaluated by analysis of variance (ANOVA) according to one or two criteria, followed by Tukey’s test, using GraphPad Instat 5.01 software (GraphPad Software Inc., San Diego, CA, USA).

### 2.9. Preclinical Trial in AD Patients

#### 2.9.1. Study Design

The study was conducted from August 2018 to March 2019 at the dermatology outpatient clinic of the Hospital das Clínicas of the Ribeirão Preto Medical School (HCFMRP-USP, Ribeirão Preto, São Paulo, Brazil). The project was approved by the Research Ethics Committee (CEP) of the School of Pharmaceutical Sciences of Ribeirão Preto (FCFRP) (CAAE 83521418.5.0000.5403). The study was a randomized, triple-blind trial conducted according to the recommendations of the CONSORT guide for clinical studies [35].

The influence of the use of cream on the need to introduce new drugs to AD control, as well as the impact of the presence of green propolis on the AD evolution, was evaluated by dividing AD patients into control (treated with CBlank) and intervention groups (treated with CPropolis). Race, sex, medications in use, and introduction or withdrawal of oral or topical medications on day 0 were considered to ensure the homogeneity of the groups.

The results were analyzed by comparing the data obtained at days 0 and 60 after using the creams.

#### 2.9.2. Study Population

The inclusion criteria were patients of both sexes diagnosed with AD, under follow-up at the dermatology outpatient clinic of the HCFMRP, aged 18 years or older, not pregnant, able to communicate verbally and understand, who did not have an allergy to any of the components of the creams, who were interested in participating in the research, and who appeared at the first consultation, even after rescheduling. As exclusion criteria, we stipulated study abandonment and the report of the lesions increasing and/or itching after using the creams.

#### 2.9.3. Sample Size Calculation

The sampling plan was performed based on calculating the sample number for experimental studies of infinite population, considering the two-tailed analysis for the alpha of 5% and a test power equal to 80% or more. The standard deviation amplitude (ΔSD) between the control and intervention groups and the estimated error value (ε) were used. The ΔSD and ε values were calculated based on the literature. For the Scoring Atopic Dermatitis (SCORAD) variable, the ΔSD and ε were the mean of 5.14 and 7.67, respectively [36,37]; as for the DLQI variable, the ΔSD and ε were 0.48 and 0.51, respectively [37]; and as for the skin hydration variable, the ΔSD and ε were 23.00 and 21.80, respectively [38]. The calculation was rotated in the statistical software MINITAB, version 17, performed for each study variable. The n calculated for SCORAD, DLQI, and hydration was, respectively, 5, 7, and 7. The largest n was used as a basis for patient selection.

#### 2.9.4. Clinical Protocol

Patients who met the inclusion criteria were invited to participate in the study after a prescheduled medical consultation at the dermatology outpatient clinic of the HCFMRP. After receiving detailed information about the research and signing the informed consent form, each patient received 10 bottles containing 200 g of cream, enough for 60 days of use.

Patients were instructed to apply a thin layer of cream twice a day to the whole body, except on the face, in the morning and evening after bathing, until return, after 60 days.

During the research, the medications prescribed and previously used by the patient were not withdrawn; they only discontinued use of any moisturizing cream already used and began to apply the cold cream received during the research period.

The clinical study was divided into three parts, with a total duration of two months. Two face-to-face meetings were held on day 0 and after 60 days, with telephone contact in the middle of the treatment after 30 days. In the face-to-face meetings, the physician evaluated the severity of the disease. The researcher evaluated skin hydration, quality of life, adherence to the use of the cream, and, in some patients, the morphology of corneocytes. Sociodemographic and clinical data were also collected. In the telephone follow-up, the researcher questioned the patient about the appearance of any adverse reaction, doubts, and adherence.

#### 2.9.5. Instruments Used in the Clinical Trial

##### Sociodemographic and Clinical Data

All research participants were registered in the study by completing an identification form (Supplementary Materials Figure S3) for sociodemographic characterization and tracing the population studied. Clinical and adherence aspects and opinions regarding the formulation characteristics were evaluated through this form. This form was prevalidated in a pilot study with six patients to verify that the questions were understood and met the study’s objective, as it had never been applied to patients with AD.

##### Scoring Atopic Dermatitis (SCORAD)

The SCORAD is a recognized instrument that measures the severity of the AD. This criterion evaluates the area of skin affected by AD, erythema, edema/papules, lichenification, xerosis, exudation/crust, and excoriation, in addition to an evaluation of the patient’s sleep quality and itching intensity. Assigned grades range from 0 to 103 points. Patients who receive a score lower than 25 have AD classified as mild. Scores between 25 and 50 characterize moderate AD, and scores higher than 50 characterize severe AD [39]. The dermatologist assigned the SCORAD in the face-to-face consultations.

##### Dermatology Life Quality Index (DLQI)

The quality of life of patients with AD was assessed using the DLQI questionnaire [40]. It measured how AD affected the patient’s life seven days before the start of the study. The items were scored ranging from zero (best health status) to 30 (worst health status), with 0–1 classified as no effect on the patient’s quality of life, 2–5 a small effect, 6–10 a moderate effect, 11–20 a substantial effect, and 21–30 a very substantial effect.

##### Evaluation of Skin Hydration

Skin surface hydration was evaluated in a delimited area on the right and left forearm with a portable digital skin analyzer (SkinUp, Brazil). The region was washed thoroughly 10 min before analysis with neutral liquid soap. Skin hydration was assessed on days 0 and 60. The mean and standard deviation of measurements in the two arms were used for comparisons.

##### Evaluation of the General Morphology of Corneocytes

The evaluation of the general morphology of the corneocytes was performed using a tape-stripping technique, with subsequent analysis of the tapes by atomic force microscopy (AFM) on days 0 and 60 [41,42]. After cleaning the region and evaluating the skin surface’s hydration, three pieces of adhesive tape (Scotch, 3M) measuring 3 × 3 cm^2^ were applied successively to the patient’s left forearm and removed after being pressed six times. The third strip removed was glued to a glass slide and analyzed by AFM to observe corneocyte morphology before and after treatment.

This technique was randomly performed in some patients, but only two samples were selected at the end of the study after the previously blind groups were revealed. Two patients with AD classified as moderate were chosen, one from the control group and the other from the intervention group. For comparison, a subject without AD was used as a control.

The corneocytes surface analysis was performed using a scanning probe microscope (SPM-9600, Shimadzu), in tapping mode (phase mode), using silicon tips (PPP-NCHR, Nanosensors), while keeping the samples under constant force conditions, at room temperature and exposed to air. The cantilever specifications adopted were: thickness—4 ± 1 μm, length—125 ± 10 μm, width—30 ± 7.5 μm, resonance frequency—204–497 kHz, force constant—10–130 N/m, and tip height—10–15 μm.

### 2.10. Statistical Analysis of the Data Obtained in the Clinical Study

The data were tabulated using Microsoft Office Excel^®^ (Office 2016, Redmond, WA, USA). The quantitative variables of quality of life, disease severity, and skin hydration had their values compared before and after treatment for each patient separately, all of them summarized using mean, standard deviation, median, minimum, and maximum.

The categorical qualitative variables were: gender, schooling, race, marital status, frequencies of consultations with the annual dermatologist, and classification of the disease severity score. The confounding variables were analyzed using absolute and relative frequencies. The association between categorical variables was evaluated using Fisher’s Exact test when there were two categories, and one of them had an n less than 5. The chi-square test per association was used for more than two categories to determine whether both groups were homogeneous regarding possible interferences to treatment. To perform Fisher’s Exact test for confounding variables, they were grouped into two categories: with and without confounding, due to the low n for each. In addition, data in 2 × 2 tables were evaluated by the McNemar test and in 3 × 2 by a Poisson model transformed to ordinal to determine whether the occurrence rate differed for both groups.

The continuous variables were compared using the *t*-test. A significance level (α) of 5% was set, and statistical analyses were conducted with the MINITAB software, version 17 (Minitab Inc., State College, PA, USA).

## 3. Results

### 3.1. Composition of Beeswax

The set of substances identified by GC/MS in the hexane, CH_2_Cl_2_-Hex, CH_2_Cl_2_-MeOH, dichloromethane/hexane, and methanol fractions of the raw and purified beeswax are presented in Table S1 and Figure S1. Both beeswaxes contained a range of aliphatic fatty acids, such as ricinoleic acid, stearic acid, and myristic acid. Purified beeswax mainly contained cerotic acid (C26) and myristyl palmitate (C46). Raw beeswax, in turn, has aromatic acids in its composition, which are not present in purified beeswax. Among the aromatic acids found in raw beeswax were cinnamic acid, *p*-coumaric acid, and ferulic acid, characteristic of green propolis [43,44].

### 3.2. Chromatographic Profiling of Raw Beeswax and Quantitative Analysis of Artepillin C

**Figure 1:**
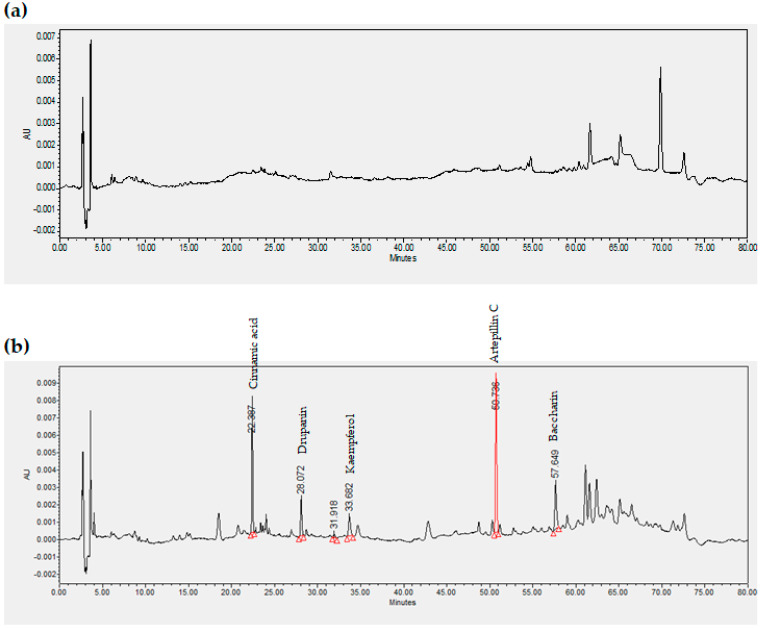

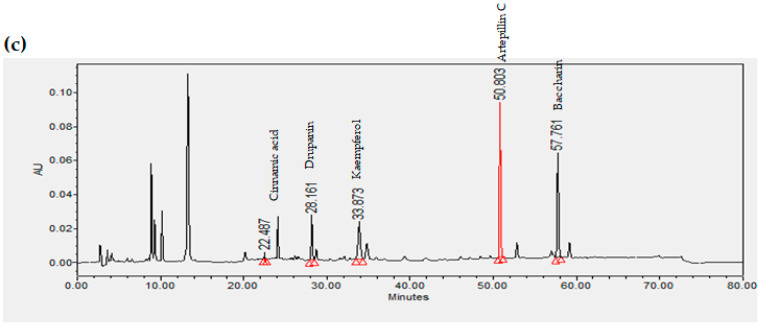
Chromatogram of concentrated methanol extract of (**a**) purified beeswax, (**b**) raw beeswax, and (**c**) green propolis. Chromatographic conditions: C18 column with a mobile phase composed of (A) water containing 0.4% formic acid, 5% methanol, and 2% isopropanol, and (B) acetonitrile and 2% isopropanol. The flow was 1 mL/min in the concentration gradient, using 20% B for 3 min, 20–25% B for 3–4 min, 25% B for 4–15 min, 25–45% B for 15–20 min, 45% B for 20–40 min, 45–60% B for 40–45 min, 60–80% B for 45–68.83 min, 80–20% B for 68.86–70 min, and 20% B for 70–80 min. An injection volume of 15 µL, oven temperature of 30 °C, and wavelength of 300 nm were used.

The chemical profile of the raw beeswax methanol fraction was evaluated by HPLC/UV to assign the main compounds of green propolis. Compound annotation was achieved by comparing the beeswax chromatogram and the standardized green propolis extract chromatogram previously chemically characterized. Figure 1 shows the chromatographic profiles of the concentrated extracts of purified beeswax, raw beeswax, and green propolis.

The raw beeswax methanol extract (Figure 1b) showed characteristic peaks of the leading green propolis extract flavonoids (Figure 1c), such as drupanin, kaempferol, artepillin C, and baccharin.

Artepillin C is the primary chemical marker for green propolis product characterization. The quantification of this compound in raw beeswax was achieved using the analytical curve of artepillin C standard (y = 0.0374x + 0.0061, r^2^ = 0.9993). The concentration of artepillin C present in raw beeswax was 20 ± 3 μg/g.

### 3.3. Anti-Inflammatory Activity of Green Propolis Extract

The different concentrations of green propolis extract evaluated, from 34 to 120 ng/mL, were not cytotoxic to the evaluated macrophage strain (Figure S2).

**Figure 2:**
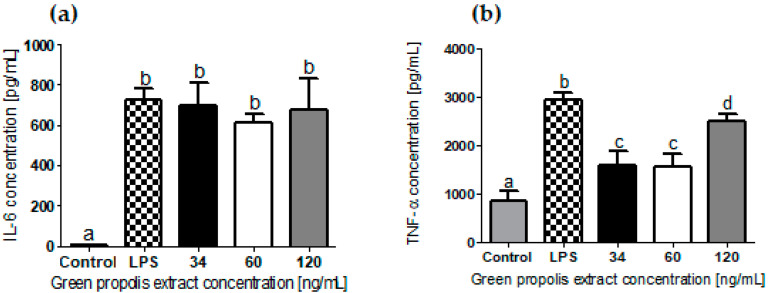
Modulation of IL-6 (**a**) and TNF-α (**b**) expression by macrophages (AMJ-2) after stimulation with LPS and treatment with green propolis extract containing artepillin C in a concentration range of 4 to 14 ng/mL. Equal letters indicate statistical similarity while different letters indicate statistical difference (ANOVA with Tukey’s post hoc test, *p* < 0.05) (*n* = 6).

The AMJ-2 macrophages did not produce significant amounts of IL-17 or INF-γ before or after stimulation with LPS. Figure 2 shows the expression of IL-6 and TNF-α by macrophages stimulated with LPS after treating them with different concentrations of green propolis extract containing from 4 to 14 ng/mL of artepillin C.

Cells stimulated with LPS significantly increased the concentration of IL-6 and TNF-α compared to nonstimulated ones (control) (ANOVA with Tukey’s post hoc test, *p* < 0.05). The cells’ treatment with propolis extract in the studied concentration range did not significantly alter the expression of IL-6. However, it significantly reduced the expression of TNF-α, mainly when concentrations lower than 120 ng/mL of propolis were used. The lowest concentrations of propolis extract studied reduced the expression of TNF-α by approximately twofold compared to cells only stimulated with LPS and not treated. Treatment with 120 ng/mL of propolis (14 ng/mL of artepillin C) reduced this expression by onefold.

### 3.4. Characterization of the Cold Creams

The creams were semisolid, homogeneous, with a white color and a light odor characteristic of fatty material. The incorporation of propolis into the cream did not perceptibly alter its odor.

In the analysis of the size of the droplets that composed the creams, determined by optical microscopy, the average number of droplets counted per quadrant was 29 ± 6 and 25 ± 6 for CBlank and CPropolis, respectively, after 1000-fold dilution. The mean droplet size was therefore similar for the two creams, at 1.8 ± 0.2 µm and 1.9 ± 0.2 µm for CBlank and CPropolis, respectively.

Table 1 shows the mechanical properties of the creams at 25 °C.

It can be seen in Table 1 that the addition of propolis extract to the cream did not significantly change the hardness, compressibility, adhesiveness, or cohesiveness. Likewise, the deformation ratio after applying a force; i.e., the elasticity, was similar (approximately 1 for the two creams).

**Figure 3:**
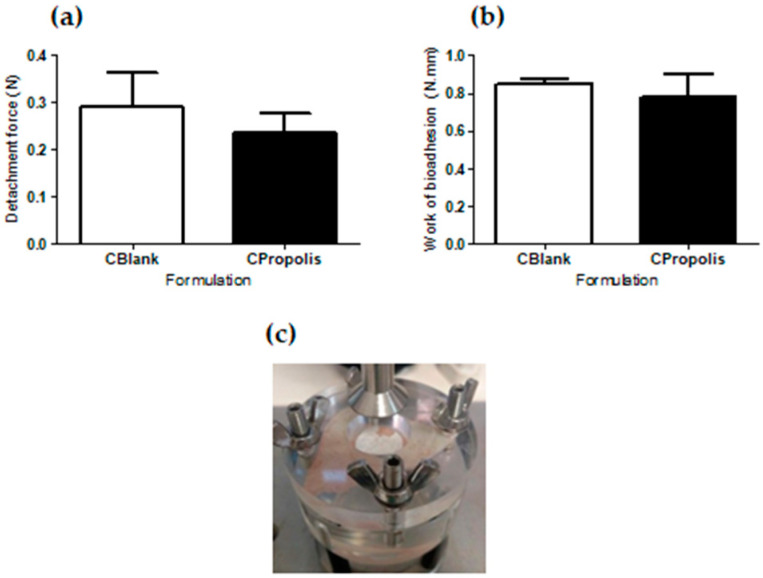
Bioadhesive properties of the creams: (**a**) detachment force; (**b**) work of bioadhesion; (**c**) representative photograph of the rupture of the bioadhesive bond between pig ear skin and cream (*t*-test, *p* > 0.05) (*n* = 5).

Figure 3 shows the detachment force, the work of bioadhesion, and the breakdown of the detachment force of the creams.

The detachment forces and work of bioadhesion of the creams were similar. Both suffered a rupture at the probe/cream interface, indicating more significant interactions between the skin and the cream [45].

### 3.5. Stability

**Figure 4:**
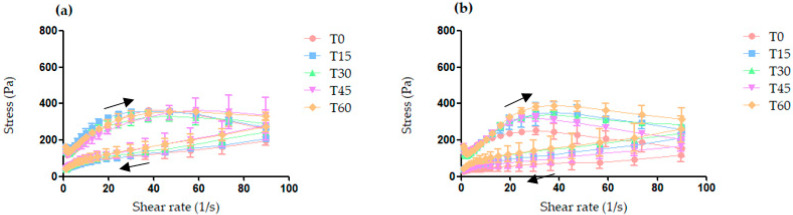
Rheological behavior of CBlank (**a**) and CPropolis (**b**) stored at room temperature, and evaluated soon after preparation (T0) and after 15 (T15), 30 (T30), 45 (T45), and 60 (T60) days. Arrows indicate the direction of the shear rate.

The storage of creams at room temperature for 60 days did not cause any apparent changes in color, odor, or physical appearance. Figure 4 shows the rheological profile of creams as a function of storage time at room temperature.

Both creams exhibited non-Newtonian pseudoplastic behavior. They also presented thixotropy and distinct ascending and descending rate curves, a phenomenon known as hysteresis.

There was no significant change in hysteresis between both creams as a function of storage time during the 60 days of study. The apparent viscosity of CBlank and CPropolis did not significantly differ for 60 and 45 days of storage, respectively (Table S2). Comparing the parameters of CBlank with CPropolis at each time, however, it was observed that CPropolis had an apparent viscosity slightly lower than CBlank at time 0 (*t*-test, *p* < 0.01), but similar at the other times.

### 3.6. Preclinical Trial in Humans

#### 3.6.1. Characteristics of the Study Population

**Figure 5:**
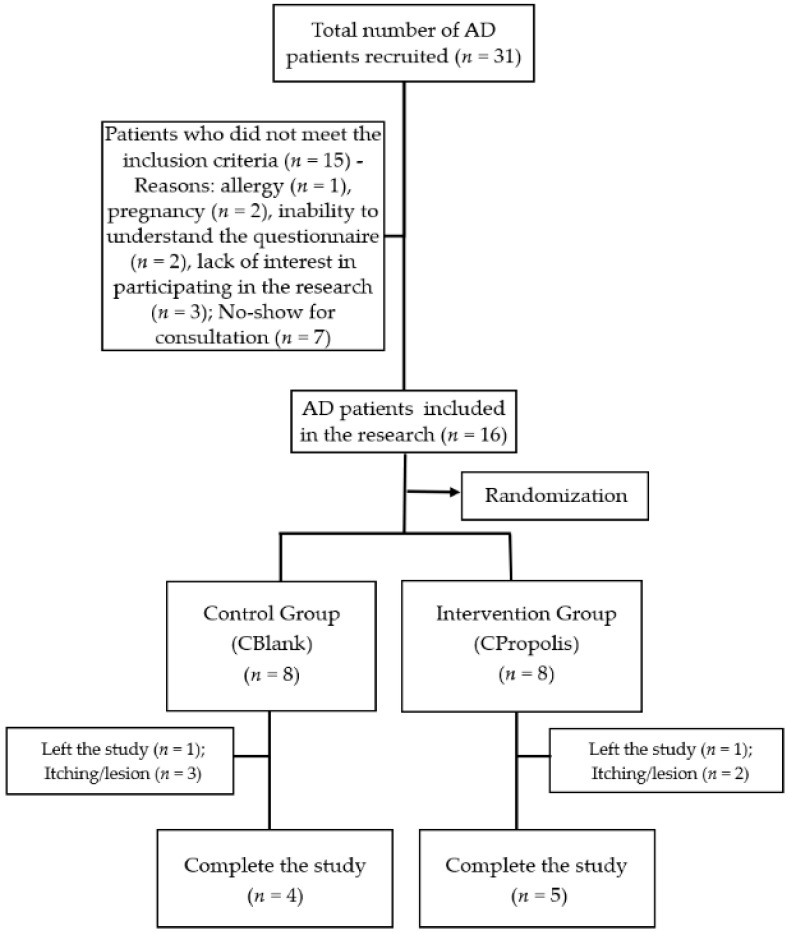
Flowchart of patient selection for the preclinical study.

From the general analysis of the medical records of patients diagnosed with AD in previous consultations, 31 patients were recruited. Figure 5 shows the flowchart that describes the excluded patients due to the inclusion criteria and the final population evaluated.

Table 2 shows the sociodemographic data of the 16 patients who were included in the study.

The mean age of patients eligible for the study was 32.5 years (SD = 11.68), ranging between 18 and 58. Regarding gender, 69% of the patients were female, and 31% were male (Table 2). Most patients saw a dermatologist up to four times a year, with an average of 3.67 ± 0.52.

For the homogeneous distribution of patients between the control and intervention groups, in addition to gender, race, and medications in use, the confounding variables of a change in the dose of methotrexate, introduction or withdrawal of oral or topical corticosteroids, the introduction of antibiotics, and a decrease in the dose of cyclosporine (Table S3) were also taken into account. Confounding variables were introduced by the physician in the first consultation after the inclusion of patients. An external researcher was responsible for the redistribution of the groups considering these variables.

In an attempt to analyze the homogeneity of the groups after distribution, the Fisher’s exact test was performed for the gender, race, and confounding variables, and the chi-square test by association for the other variables. The *p*-value was more significant than 0.05 for all variables considered for the group’s randomization, proving that the groups were evenly distributed.

It was possible to observe that most patients in both the control and intervention groups had allergic diseases, but the patients who suffered from them were also homogeneously distributed (Table S4).

As there were no significant differences between the mechanical (Table 1) and bioadhesive (Figure 3) properties of the creams, adhesion aspects were treated together. Fear of side effects, unpleasant smell, itchiness, and appearance change was reported by only one patient. Three patients reported that the cream was difficult to spread. A high percentage of patients, 89%, reported that the cream made their skin oily. This aspect was, however, considered positive for seven of the eight patients who reported it.

The intervention and control group’s adherence and acceptability were similar, as the number of patients who dropped out of the study was the same in the two groups (one patient in each group).

#### 3.6.2. Evaluation of SCORAD, DLQI, and Skin Hydration

Table 3, Table 4 and Table 5 show the results of SCORAD, DLQI, and skin hydration, respectively, obtained at the beginning of the study and after 60 days of using the creams.

In general, comparing the means obtained from SCORAD, DLQI, and skin hydration before and after 60 days of treatment, no significant change (*p* > 0.05) in the values and classifications derived from them was observed.

Attention was drawn to the DLQI values (Table 4), which, despite the absence of a statistical difference at the beginning of treatment, were lower for all patients studied after 60 days. This result suggests the disease had a lower impact on the patient’s quality of life using the creams.

The analysis of individual SCORAD values (Table 3) was also lower after 60 days for most patients who used CPropolis.

Table 6 and Table 7 show the impact of using CBlank and CPropolis, respectively, on SCORAD, DLQI, and skin hydration for each patient. They were plotted to facilitate the analysis of these three variable’s impact on the patient’s individual clinical status.

Of the patients who used CBlank (Table 6), only patient 2 had confounding variables: the introduction of oral antibiotics and topical corticosteroids. In this patient, the use of the cream did not change the parameters analyzed; that is, there was no exacerbation of the disease, which may have been the effect of using CBlank or the introduction of new treatments. However, for patients who did not have confounding variables, there was an increase in the classification of disease severity and decreased hydration for patients 1 and 4, but an improvement in quality of life. For patient 4, this improvement was quite significant, with DLQI before using the cream classified as “very large” and reaching the classification “no effect” after 60 days of use. For patient 3, the use of the cream did not change the classification of the disease and skin hydration; however, the quality of life improved, suggesting that its use could control AD, preventing its exacerbation.

In the patients who used CPropolis (Table 7), the vast majority had decreased SCORAD values. Patient 1, who did not present confounding variables, presented a change in the classification of the disease, from severe to moderate, in addition to a significant improvement in quality of life and skin hydration. Except for patient 2, who had severe AD and presented introducing oral corticosteroids as a confounding variable, all patients reported an improvement in their quality of life. Patient 3 had no confounding variable and had reduced skin hydration, but SCORAD was maintained, and quality of life improved. Patients 4 and 5, who had reduced dosage of cyclosporine and withdrawal of oral corticosteroids, which could worsen the AD, did not show changes in the classification of disease severity, and showed improvement in skin hydration and DLQI.

Furthermore, during the final interview, 80% of the patients in the intervention group reported that the cream reduced itching and increased skin hydration more effectively than other creams previously used. In the control group, only one patient (25%) reported a similar fact.

#### 3.6.3. Assessment of General Corneocyte Morphology

**Figure 6:**
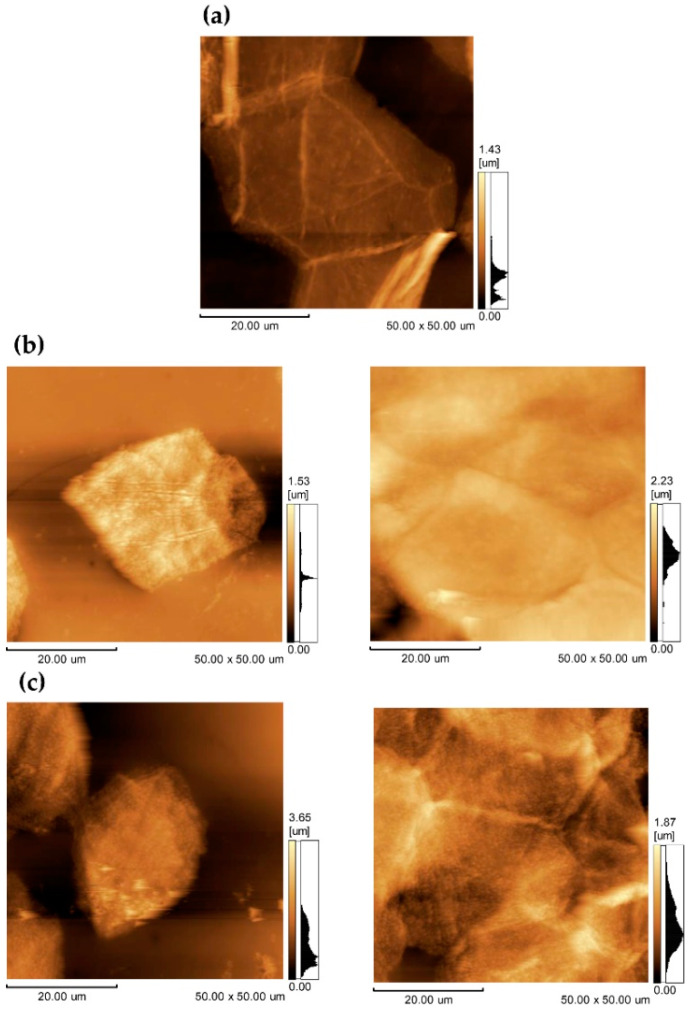
Photomicrographs obtained by AFM, in tapping phase mode, of adhesive tapes containing fragments of the stratum corneum from a healthy patient (**a**), from a patient with moderate AD who used the CBlank cream (**b**), and from a patient with moderate AD who used the CPropolis cream (**c**). The images of AD patients show the stratum corneum before treatment in the left panel and after 60 days of treatment in the right panel.

Figure 6 shows AFM images of skin corneocytes from a healthy individual; from patient 3, who used CBlank; and patient 1, who used CPropolis, before and after 60 days of using the cream.

In the image of the stratum corneum of the individual’s skin without AD (Figure 6a), more than one corneocyte can be seen joining another per field, which had a hexagonal shape and flat topography [41,46]. On the other hand, the images of the stratum corneum of patients with AD before using the cream (Figure 6b,c, left panel) show isolated corneocytes with a different shape from the healthy cell. Treatment with creams for 60 days (Figure 6b,c, right panel), regardless of the presence of propolis, allowed the observation of more corneocytes per field, surrounded by others, and with a shape closer to the normal hexagonal.

## 4. Discussion

It is known that the use of moisturizers, especially those with high oil content, such as cold cream, is an essential measure for the treatment of patients with AD. They usually improve skin emollience and hydration, and avoid skin sensitization to various stimuli [4].

Purified beeswax, a primordial component of cold cream, presents in its composition a range of aliphatic fatty acids, among them stearic, myristic, and ricinoleic acids, in addition to cerotic acid and merissyl palmitate (Table S1). Thus, they have great potential to help restore the stratum corneum deficient in long-chain fatty acids in patients with AD [47].

In clinical practice, the use of cold cream made with raw beeswax, in place of traditional purified beeswax, showed greater adherence of patients to treatment. However, raw beeswax certainly is not recommended for the large-scale production of cold cream, due mainly to raw material quality-control problems. Therefore, preparing stable and reproducible cold cream is an essential step in developing formulations for AD control.

The first part of this work consisted of verifying the hypothesis that raw beeswax would have components of propolis not present in purified beeswax. Once this hypothesis was confirmed, in a second stage, cold cream was obtained with purified beeswax and supplemented with known amounts of green propolis for a randomized, double-blind study in AD patients.

The patterns of fragmentation of fatty compounds present in the different fractions of raw and purified beeswaxes revealed that raw beeswax, different from purified ones, had aromatic acids such as cinnamic acid, p-coumaric acid, and ferulic acid. These acids are characteristic of green propolis [43,44] and have anti-inflammatory and antioxidant properties [48]. The comparative analysis of raw beeswax extract, which presented these aromatic compounds with a standardized extract of green propolis, confirmed green propolis markers in raw beeswax (Figure 1).

The anti-inflammatory potential of propolis in the concentration range of 5 to 20 µg/mL was recently evidenced by in vitro experiments in which LPS-induced TNF-α and IL-6 levels were reduced when exposed to Brazilian propolis extract [49]. However, the amount of propolis present in raw beeswax is small, and only around 15% of beeswax makes up cold creams. In addition, the stratum corneum hampers the penetration of substances into the skin. Thus, the anti-inflammatory potential of propolis concentrations in the nanogram range was evaluated in vitro in a culture of macrophages stimulated with LPS (Figure 2).

A significant reduction in the expression of TNF-α was observed using 34 to 120 ng/mL of green propolis extract. Surprisingly, the reduction was higher at the lower concentrations of 34 and 60 ng/mL. It is known that there is a dynamic balance in the anti- and proinflammatory mediator’s expression that changes in the face of aggression [50]. It is possible that at concentrations greater than 60 ng/mL, the various components present in propolis extract stimulate the production of other inflammatory mediators not monitored in the present study, reducing the effect on TNF-α expression. Nevertheless, 120 ng/mL of propolis also was able to decrease the expression of TNF-α in the control stimulated only with LPS, suggesting that propolis can modulate its expression even at low concentrations. TNF-α alone or in combination with Th2 cytokines decreases the levels of long-chain free fatty acids and specific ceramides, affecting the organization of the lipid matrix of the stratum corneum [51]. The modulation of TNF-α expression induced by green propolis may therefore be beneficial for the control of AD.

The addition of propolis extract to cold cream did not alter its mechanical characteristics (Table 1) or bioadhesive properties (Figure 3). They also remained stable and had similar rheological behavior for at least 60 days of storage. The only notable difference between the two creams was that the initial apparent viscosity of CPropolis was slightly lower than that of CBlank. The addition of green propolis extract, characterized by a complex mixture of substances, including the flavonoids highlighted in Figure 1, seems to have modified the interactions between the droplets, resulting in a decrease in viscosity [52]. After 15 days, however, no differences were found between the creams (Table S2), suggesting the need for a longer time for the dispersion to reach the equilibrium when propolis is added.

Interestingly, the cold cream showed a detachment force three times higher than a 6% chitosan hydrogel, a polymer with known mucoadhesive properties [53]. In addition, the rupture of bioadhesive bonding of the creams occurred at the interface between the cream and the probe (Figure 3c), suggesting a greater force of interaction with the skin, an essential feature for the cream to remain adhered to the patient’s skin after application.

The clinical study was mainly carried out to evaluate the influence of low concentrations of green propolis on AD control. Green propolis is usually added in creams at concentrations of 2% to 10% [54,55,56], at least 1000 times higher than in CPropolis. High concentrations of propolis leave a characteristic odor in creams that can stimulate allergic processes in AD patients [57]. In addition, the possibility of propolis having an anti-inflammatory effect more pronounced in low concentrations, as demonstrated in vitro (Figure 2), is advantageous for AD treatment.

Of the 16 patients included in the study, 5 (31%) were excluded due to the reported appearance of new lesions and skin itching (Figure 5). These reactions may have been caused by the cream’s allergic reaction or other uncontrolled factors, such as exposure to other allergens or the patient’s psychological balance. For example, some of the patients who presented skin lesions reported that they were stressed, a factor that contributes to the triggering of AD lesions [58]. However, only 12% of patients who reported new lesions and pruritus were from the intervention group, against 19% from the control group. This distribution suggests that the green propolis extract added to the cream was not the cause of the appearance of lesions and itching. On the contrary, the results suggest that propolis prevented or decreased the appearance of pruritus due to its anti-inflammatory activity. This improvement corroborates the results obtained in the in vitro anti-inflammatory assay (Figure 2).

Due to the variability of the initial health status of each patient and confounding variables, it was not possible to observe statistically significant differences between the means of SCORAD, DLQI, and skin hydration before and after 60 days of treatment. Therefore, it can be generally inferred that the creams could control the evolution of the disease, avoiding the introduction of new drugs in the AD treatment.

Furthermore, allied to the peculiar clinical status of each patient before the beginning of study, no medication in use was withdrawn due to ethical issues. However, the doctor had the freedom to withdraw or introduce medications in the first consultation performed immediately after the inclusion of the patient in the study. These confounding variables were considered punctually for a better accurate understanding of the clinical impact of creams. Thus, although the control and intervention groups were homogeneous, the individual analysis of the patients allowed a more accurate assessment of the clinical status of the same during the use of the cream, considering individual variables.

Based on the individual analysis of the control group (Table 6), it can be stated that the use of CBlank improved how the patient dealt with the disease, increasing their quality of life. Its use did not appear, however, to have interfered with the course of AD.

The use of CPropolis, on the other hand, seems to have been a better alternative than CBlank to avoid the sharpening of AD (Table 7). Both creams, however, improved the quality of life of patients, an essential point for emotional and disease control.

However, it should be emphasized that the clinical study in humans was an initial study. In future studies, for a more accurate evaluation of the differences between creams, a more significant number of patients participating in the study may be necessary. Although the number of patients evaluated followed that obtained from the sample calculation, this was based on data obtained from the literature for experiments carried out with conventional drugs added to creams. Studies evaluating only the effect of the base cream on AD, as performed in this study, were not found.

The analysis of the morphology of corneocytes after tape stripping by AFM was performed only as a preliminary study to glimpse the possibility of, in future studies, evaluating the influence of formulations on the skin of patients with AD in a noninvasive way. The technique allowed the adequate visualization of the corneocytes (Figure 6). In addition, an improvement of AD patient’s corneocyte morphology could be noticed after cream treatment.

In summary, low ng/mL propolis concentrations can reduce TNF-α expression, overexpressed in the skin of patients with AD. Incorporation into a stable cold cream did not alter its mechanical and rheological characteristics. When applied to patients with AD for 60 days, cold cream with propolis seemed to prevent the disease from getting worse, and improved patient life quality.

## Figures and Tables

**Table 1:** Mechanical properties of CBlank and CPropolis at 25 °C.

Mechanical Properties	CBlank	CPropolis
Hardness (×10^−1^ N)	4.9 ± 0.1	5.2 ± 0.1
Compressibility (×10^−1^ N.mm)	9.0 ± 0.3	9.5 ± 0.5
Adhesiveness (×10^−1^ N.mm)	5.4 ± 0.2	5.7 ± 0.2
Cohesiveness	6.5 ± 0.1	6.6 ± 0.1

Values expressed as mean ±standard deviation (SD). There was no statistical difference between the creams for any of the analyses (*t*-test, *p* < 0.05) (*n* = 10).

**Table 2:** Sociodemographic data and access to the patients’ health service.

Population	Control Group (*n* = 8)	Frequency (%)	Intervention Group (*n* = 8)	Frequency (%)	*p*-Value
**Gender**					
Female	5	62.5	6	75	0.999 *
Male	3	37.5	2	25	
**Race**					
White	7	87.5	6	75	0.999 *
Black	1	12.5	2	25	
**Marital Status**					
Married	4	50	3	37.5	0.120
Single	2	25	5	62.5	
Divorced	2	25	0	0	
**Education Level**					
Incomplete Elementary School	1	12.5	1	12.5	1.000
Elementary School	1	12.5	0	0	
Middle School	5	62.5	6	75	
Higher Education	1	12.5	1	12.5	
**Frequency of visits to the** **dermatologist (Times/Year)**					
0–4	6	75	4	50	0.999
5–7	1	12.5	1	12.5	
8–12	1	12.5	3	37.5	

* p-value calculated by Fisher’s exact test. The chi-square test was used to calculate the *p* of the other variables.

**Table 3:** Disease severity score (SCORAD) at the beginning of the study and after 60 days of treatment with cold creams CBlank (Control) and CPropolis (Intervention).

Patient	Control Group				Intervention Group			
	Outset		60 Days		Outset		60 Days	
	Points	Classification	Points	Classification	Points	Classification	Points	Classification
1	42.0	Moderate	52.5	Severe	65.0	Severe	42.0	Moderate
2	47.2	Moderate	32.5	Moderate	62.5	Severe	58.5	Severe
3	45.5	Moderate	49.0	Moderate	12.4	Mild	12.9	Mild
4	13.0	Mild	25.1	Moderate	29.0	Moderate	32.5	Moderate
5	-	-	-	-	21.5	Mild	19.0	Mild
Mean ± SD	37 ± 16		40 ± 13		38 ± 24		33 ± 18	

Start of statistical analysis × 60 days: Points: *t*-test Control *p*-value: 0.674; Intervention: 0.175. Classification: Control: McNemar test, *p*-value: 0.705; Intervention: Model of Poisson, *p*-value: 0.739. Control 60 days × Intervention 60 days: Points: *t*-test, *p*-value: 0.5526.

**Table 4:** Effect on the patient’s quality of life (DLQI) of treatment with cold creams CBlank (Control) and CPropolis (Intervention) for 60 days.

Patient	Control Group				Intervention Group			
	Outset		60 Days		Outset		60 Days	
	Points	Effect on DLQI	Points	Effect on DLQI	Points	Effect on DLQI	Points	Effect on DLQI
1	2	Small	1	No effect	15	Very large	1	No effect
2	8	Moderate	6	Moderate	17	Very large	16	Very large
3	15	Very large	7	Moderate	8	Moderate	3	Small
4	15	Very large	1	No effect	10	Moderate	4	Small
5	-	-	-	-	5	Small	0	No effect
Mean ± SD	10 ± 6		4 ± 3		11 ± 5		5 ± 6	

QL = Quality of life. Start of statistical analysis × 60 days: Points, *t*-test: *p*-value Control: 0.129; *p*-value Intervention: 0.051. Control 60 days × Intervention 60 days: Points: *t*-test: *p*-value: 0.7768.

**Table 5:** Effect on skin hydration of treatment with cold creams CBlank (Control) and CPropolis (Intervention) for 60 days.

Patient	Control Group		Intervention Group	
	Outset	60 Days	Outset	60 Days
1	28	14	18	27
2	39	31	46	48
3	58	56	86	36
4	44	27	16	30
5	-	-	51	45
Mean ± SD	42 ± 12	32 ± 17	43 ± 29	37 ± 9

Start of statistical analysis × 60 days: *t*-test: Control *p*-value: 0.055; Intervention *p*-value: 0.665. Control 60 days × Intervention 60 days: *t*-test: *p*-value: 0.5816.

**Table 6:** SCORAD, DLQI, and skin hydration of each patient treated with CBlank (Control).

Variable	Patient			
	Patient 1		Patient 2	
	Outset	After 60 Days	Outset	After 60 Days
SCORAD	42 (moderate)	52.5 (severe)	47.2 (moderate)	32.5 (moderate)
DLQI	2 (small)	1 (no effect)	8 (moderate)	6 (moderate)
Hydration	28	14	39	31
**Variable**	**Patient**			
	**Patient 3**		**Patient 4**	
	**Outset**	**After 60 Days**	**Outset**	**After 60 Days**
SCORAD	45.5 (moderate)	49 (moderate)	13 (mild)	25.1 (moderate)
DLQI	15 (very large)	7 (moderate)	15 (very large)	1 (no effect)
Hydration	58	56	44	27

**Table 7:** SCORAD, DLQI, and skin hydration of each patient treated with CPropolis (Intervention).

Variable	Patient			
	Patient 1		Patient 2	
	Outset	After 60 Days	Outset	After 60 Days
SCORAD	65 (severe)	42 (moderate)	62.5 (severe)	58.5 (severe)
DLQI	15 (very large)	1 (no effect)	17 (very large)	16 (very large)
Hydration	18	27	46	48
**Variable**	**Patient 3**		**Patient 4**	
	**Outset**	**After 60 Days**	**Outset**	**After 60 Days**
SCORAD	12.4 (mild)	12.9 (mild)	29 (moderate)	32.5 (moderate)
DLQI	8 (moderate)	3 (small)	10 (moderate)	4 (small)
Hydration	86	36	16	30
**Variable**	**Patient 5**			
	**Outset**	**Outset**		
SCORAD	21.5 (mild)	21.5 (mild)		
DLQI	5 (small)	5 (small)		
Hydration	51	51		

## Data Availability

The data presented in this study are available on request from the Corresponding Author.

## Supplementary Materials

The following are available online at https://www.mdpi.com/article/10.3390/pharmaceutics13091346/s1. Figure S1: Chromatograms of raw and purified beeswax samples in the different fractions studied, obtained by GC/MS. Chromatograms of fractions: (a) methanol, (b) hexane, (c) dichloromethane/hexane, (d) CH_2_Cl_2_-MeOH, and (e) CH_2_Cl_2_-Hex. Chromatographic conditions: Helium gas pressure of 187.1 kPa, linear viscosity of 31.9 cm/s, and column flow of 1.5 mL/min. Ion source temperature: 250 °C; mass range: 40 to 700 *m*/*z* every 0.3 s. Databases: NIST11, NIST11-S, WILEY7, NIST08, and FFNSC3.1. Figure S2: Percentage of viable cells (AMJ-2) after treatments with different concentrations of green propolis extract. Table S1: Substances in raw and purified beeswax. Analyses were carried out by GC/MS, and annotation was based on the similarity of theoretical spectra found in the NIST11, NIST11-S, WILEY7, NIST08, and FFNSC3.1 databases. Table S2: Apparent viscosity (30 s) and area of hysteresis of the creams as a function of storage time at room temperature. Table S3: Confounding variables taken into account in the distribution of groups. Table S4: Allergic comorbidities presented by patients included in the study. Figure S3: Sociodemographic and clinical data.
