# Supplementary material for: A New Approach to Atopic Dermatitis Control with Low-Concentration Propolis-Loaded Cold Cream
Source: Pharmaceutics. 2021 Aug 27;13(9):1346. doi: 10.3390/pharmaceutics13091346 (PMC8466707; doi:10.3390/pharmaceutics13091346)
Supplement: Supplementary file 1 [file pharmaceutics-13-01346-s001.zip › pharmaceutics-1290073-supplementary.pdf]

pharmaceutics

MDPI

Supplementary Materials: A New Approach to Atopic Dermatitis Control with Low-Concentration Propolis-Loaded Cold Cream

Bianca Aparecida Martin, Camila Nunes Lemos, Luciana Facco Dalmolin, Caroline Arruda, Íris Sperchi Camilo Brait, Maurilio de Souza Cazarim, Estael Luzia Coelho da Cruz-Cazarim, Paula Carolina Pires Bueno, Maurílio Polizello Júnior, Leonardo Régis Leira Pereira, Renata Nahas Cardili and Renata Fonseca Vianna Lopez

(a)	
**Methanol:Water Fraction**	
**Raw Besswax**	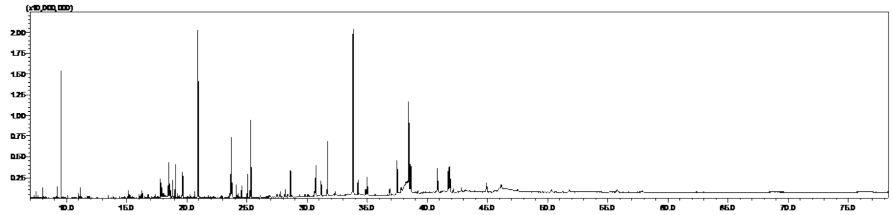
**Purified Besswax**	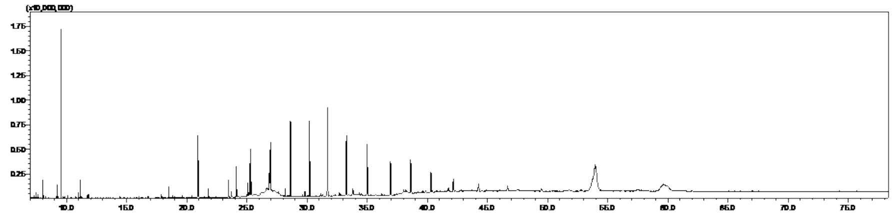
**(b)**	
**Hexane Fraction**	
**Raw Besswax**	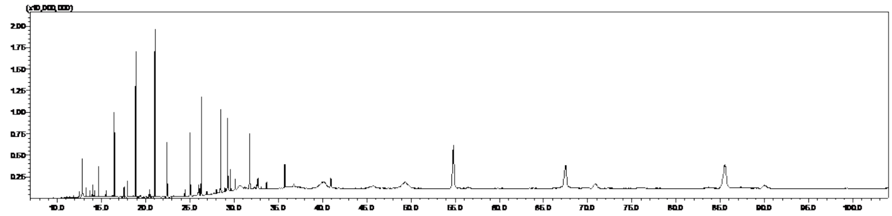
**Purified Besswax**	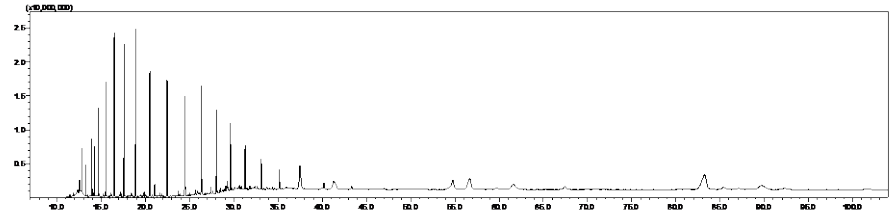

(c)

Dichloromethane:Hexane Fraction

Raw Besswax

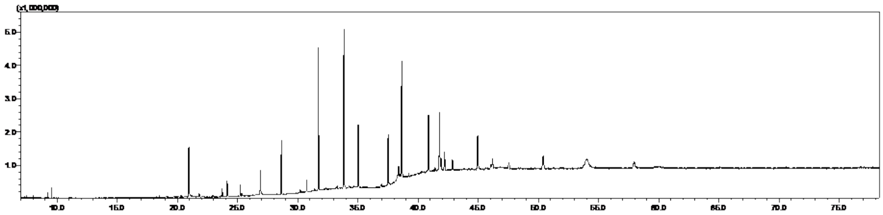

Purified Besswax

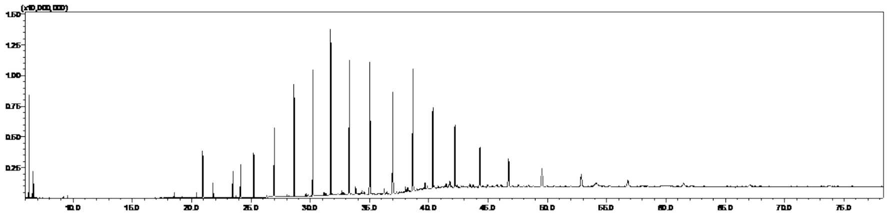

(d)

Dichloromethane-M Fraction

Raw Besswax

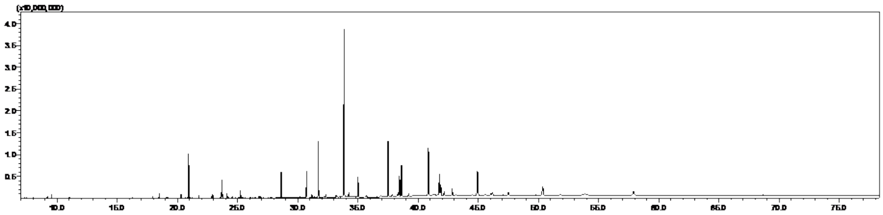

Purified Besswax

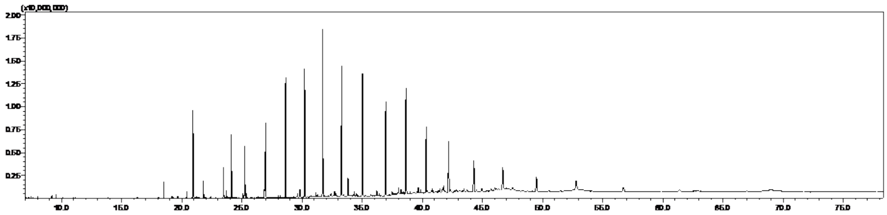

(e)

Dichloromethane-H Fraction

Raw Besswax

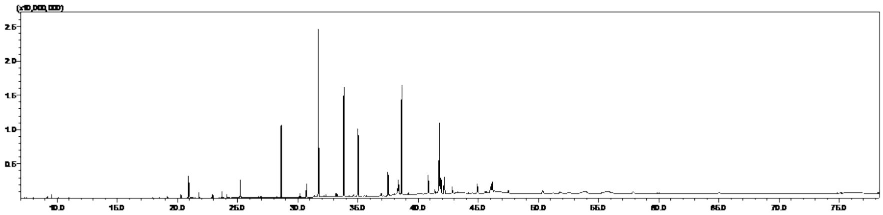

Purified Besswax

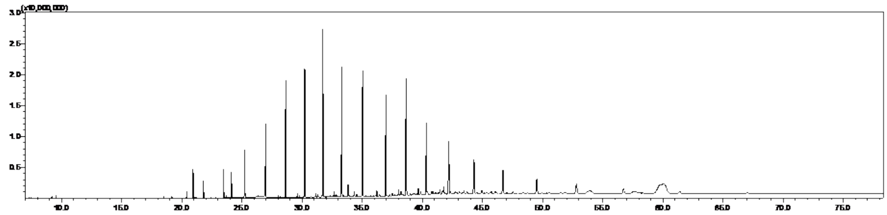

Figure S1. Chromatograms of samples of raw and purified beeswax in the different fractions studied, obtained by GC/MS. Chromatograms of fractions (**a**) methanol, (**b**) hexane, (**c**) dichloromethane:hexane, (**d**) CH_2Cl_2-MeOH and (**e**) CH_2Cl_2-Hex. Chromatografic conditions: Helium gas pressure of 187.1 kPa, linear viscosity 31.9 cm/s and column flow 1.5 mL/min. Ion source temperature 250°C and mass range 40 to 700 *m/z* every 0.3 s. Database: NIST11, NIST11-S, WILEY7, NIST08 and FFNSC3.1.

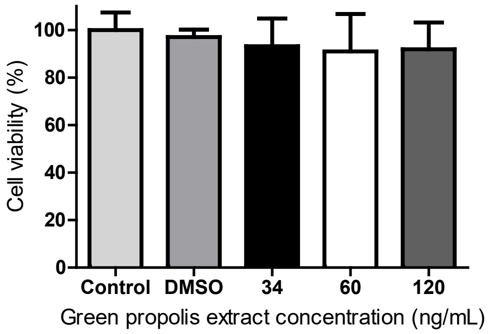

Figure S2. Percentage of viable cells (AMJ-2) after treatments with different concentrations of green propolis extract (ANOVA with Tukey's post hoc test, $p > 0.05$).

Table S1. Substances in raw and purified beeswax. Analyses were carried out by GC/MS and annotation was based on the similarity of theoretical spectra found in the NIST11, NIST11-S, WILEY7, NIST08 and FFNSC3.1 databases.

		Samples										
				RT				Fraction (Extraction)				
N°	Components *	*Raw Besswax*	*Purified Besswax*	(min)	Database	SI	Metabolic Class	Methanol:Water	Dichloromethane M	Dichloromethane H	Hexane	Dichloromethane:Hexane
1	(Z) 3-Phenyl-2-propenoic acid ou Cinnamic acid (1TMS) *	x	-	15.16	NIST11s	95	Phenylpropanoids	x				
2	1,3-Dipalmitin (1TMS)	-	x	43.72	NIST11s	69	Glycerolipid				x	
3	1,9- Nonanedioic acid ou Azelaic acid (2TMS)	x	x	18.01	NIST11s	92	Fatty acid	x				
4	2-Ethylbutyric acid, nonadecyl ester	-	x	41.31	NIST08	79	Fatty acid				x	
5	4-Hydroxy-3,5,5-trimethyl-4-[3-oxo-1-butenyl]-2-cyclohexen-1-one	x	-	18.10	NIST11s	93	Ketone	x				
6	4-Hydroxy-3-methoxy benzoic acid ou Vanillic acid (2TMS)	x	x	17.57	WILEY7	85	Benzene and substituted derivatives	x				
7	4-Hydroxy-3-methoxy cinnamic acid ou Ferulic acid (1TMS) *	x	-	21.62	NIST08	83	Phenylpropanoids	x				
8	4-methoxy-benzeneacetic acid (1TMS)	x	-	15.04	NIST11	86	Benzene and substituted derivatives	x				
9	4-Methoxy-benzoic acid (1TMS)	x	x	14.81	NIST11	92	Benzene and substituted derivatives	x				
10	9,12-Octadecadienoic acid (Z,Z) (1TMS)	x	-	23.60	WILEY7	96	Fatty acid	x	x	x		

11	9,12-Octadecadienoic acid, ethyl ester	x	-	13.71	WILEY7	82	Fatty acid				x	
12	9-Octadecadienoic acid, ethyl ester	x	-	22.91	NIST11	94	Fatty acid	x				
13	9-Octadecenoic acid (1TMS)	x	-	14.05	WILEY7	96	Fatty acid				x	
14	9-Octadecenoic acid, ethyl ester	x	-	22.92	NIST11	95	Fatty acid			x	x	
15	beta.-L-Mannofuranose, 6-deoxy-1,2,3,5-tetrakis-O-(1TMS)-?	x	-	18.21	WILEY7	88	Sugar	x				
16	Butanedioic acid (2TMS)	-	x	11.81	WILEY7	95	Carboxylic acid and derivatives	x				
17	D-(-)-Tagatofuranose, pentakis, (1TMS) ether (isomer 1)?	x	-	17.88	NIST11	91	Sugar	x				
18	D-(+)-Talofuranose, pentakis, (1TMS) ether (isomer 1)?	x	-	18.17	NIST11	87	Sugar	x				
19	Decanedioic acid ou Sebacic acid (2TMS)	-	x	18.96	NIST08	92	Fatty acid	x				
20	Docosane	-	x	23.45	FFNSC1.3	97	Hydrocarbon	x	x	x	x	x
21	Docosanoic acid (1TMS)	x	-	30.71	NIST11s	95	Fatty acid	x	x	x		x
22	Dodecanoic acid (1TMS)	x	-	16.28	WILEY7	94	Fatty acid	x				
23	Dotriacontanoic acid (1TMS)	x	-	50.36	NIST08	86	Fatty acid		x	x	x	x
24	Dotriacontanol (1TMS)	x	-	47.50	NIST11	95	Fatty acid		x	x	x	x
25	D-Psicofuranose, pentakis, (1TMS) ether (isomer 2)?	x	-	17.80	NIST11	91	Sugar	x				
26	Eicosane	x	x	20.38	FFNSC1.3	97	Hydrocarbon	x	x	x	x	x
27	Glucopyranose, pentakis-O (1TMS)?	x	-	19.24	NIST11	94	Sugar	x				
28	Glyceric acid (3TMS)	x	-	11.94	NIST11s	90	Sugar	x				
29	Hecacosyl hexadecanoate	x	-	54.00	NIST11	88	Fatty acid					x
30	Hentriacontane	x	x	38.61	FFNSC1.3	97	Hydrocarbon		x			
31	Heptacosane	x	x	31.70	FFNSC1.3	97	Hydrocarbon		x			
32	Heptadecanoic acid (1TMS)	-	x	22.39	NIST11s	92	Fatty acid		x			
33	Heptanoic acid, docosyl ester	-	x	87.15	WILEY7	69	Fatty acid				x	
34	Heptatriacontane	-	x	52.80	NIST08	96	Fatty acid		x	x		x
35	Hexacosane	x	x	30.20	NIST11	97	Hydrocarbon				x	
36	Hexacosanoic acid (1TMS)	x	x	37.48	NIST08	96	Fatty acid		x	x	x	x
37	Hexacosanol (1TMS) ether	x	-	35.71	NIST11	84	Fatty acid		x			
38	Hexadecanoic acid (1TMS)	x	x	20.66	WILEY7	96	Fatty acid	x				
39	Hexadecanoic acid, eicosyl ester	-	x	92.17	NIST08	79	Fatty acid				x	
40	Hexadecanoic acid, ethyl ester	x	-	20.29	WILEY7	96	Fatty acid		x	x		
41	Hexadecanoic acid, ethyl ester, ethyl palmitate	x	-	20.28	WILEY7	95	Fatty acid	x				
42	Hexadecanoic acid, octacosyl ester	x	-	85.48	WILEY7	89	Fatty acid				x	
43	Hexadecanoic acid, propyl ester (2TMS)	x	x	29.80	NIST11s	93	Fatty acid	x				
44	Hexadecenoic acid (1TMS)	x	x	20.67	NIST11	92	Fatty acid	x	x			

45	Hexatriacontane	-	x	49.50	NIST08	97	Hydrocarbon	x	x	x	x	x
46	Hexatriacontanoic acid (1TMS)	x	-	68.59	NIST08	78	Hydrocarbon		x			
47	Hydrocinnamic acid ou benzenepropanoic acid (1TMS)	x	x	13.44	NIST11	93	Phenylpropanoids	x				
48	Hydroxicinnamic acid ou p-Coumaric acid (1TMS) *	x	-	19.66	WILEY7	87	Phenylpropanoids	x				
49	Monopalmitin (1TMS) ether	-	x	32.75	NIST11s	93	Glycerolipid	x				
50	Nonacosane ou n-Nonacosase	x	x	35.35	FFNSC1.3	97	Hydrocarbon	x	x	x		x
51	Nonadecane ou n-Nonadecane	x	-	19.17	WILEY7	97	Hydrocarbon		x	x	x	x
52	Nonatriacontane	-	x	61.40	WILEY7	97	Hydrocarbon		x			x
53	Octacosane	x	x	33.27	FFNSC1.3	97	Fatty acid		x			
54	Octacosanoic acid (1TMS)	x	x	40.80	NIST08	89	Fatty acid	x	x			x
55	Octacosanol (1TMS)	x	-	39.19	NIST11s	97	Fatty acid		x		x	x
56	Octadecanoic acid ou stearic acid (1TMS)	x	x	24.12	NIST11s	95	Fatty acid	x	x	x		x
57	Octadecenoic acid (1TMS)	x	x	23.69	WILEY7	96	Fatty acid	x	x			x
58	Octadecenoic acid, ethyl ester	x	-	22.90	NIST11	94	Fatty acid	x				
59	Octatriacontane	-	x	56.70	NIST08	96	Fatty acid		x	x		x
60	Pentacosane	x	x	28.61	FFNSC1.3	97	Hydrocarbon	x	x	x		x
61	Pentatriacontane	-	x	46.66	NIST11s	92	Hydrocarbon	x	x	x		x
62	p-Hydroxybenzoic acid (1TMS)	x	x	16.02	NIST11s	90	Benzene and substituted derivatives	x				
63	Ricinoleic acid (1TMS)	x	x	26.87	WILEY7	93	Fatty acid	x	x	x		x
64	Tetracontane	-	x	67.00	FFNSC1.3	83	Hydrocarbon		x			x
65	Tetracontanoic acid (1TMS)	x	-	40.94	NIST08	85	Fatty acid				x	
66	Tetracosan-1-ol (1TMS) ether	x	-	32.31	NIST08	81	Fatty acid		x	x		
67	Tetracosane	x	x	26.95	WILEY7	97	Hydrocarbon	x	x	x		x
68	Tetracosanoic acid (1TMS)	x	x	33.80	NIST11	95	Fatty acid	x	x	x		x
69	Tetracosanoic acid, ethyl ester	x	-	33.18	FFNSC1.3	93	Fatty acid		x			
70	Tetracosanol (1TMS) ether	x	-	32.32	NIST08	81	Hydrocarbon		x			
71	Tetradecanoato, ethyl	x	-	33.17	FFNSC1.3	94	Hydrocarbon			x		
72	Tetradecanoic acid ou myristic acid (1TMS)	x	x	18.50	WILEY7	96	Fatty acid	x	x	x		x
73	Tetratriacontane	-	x	44.25	NIST11	97	Hydrocarbon	x	x	x		x
74	Triacontane	x	x	36.95	FFNSC1.3	97	Hydrocarbon	x		x		x
75	Triacontanoic acid (1TMS)	x	-	44.91	WILEY7	86	Fatty acid	x	x	x		x
76	Triacontanol (1TMS) ether	x	-	42.82	NIST11	89	Fatty acid		x	x		x
77	Tricosane ou n-Tricosane	x	x	25.21	WILEY7	97	Hydrocarbon	x	x	x		x
78	Tritriacontadiene	x	-	41.39	NIST11	94	Hydrocarbon			x		

* Shadow compounds are aromatic substances found in green propolis. TMS: Substances identified in the databases as silylated derivatives. RT: Average retention time considering all values found in the different fractions. SI: Similarity index. NOTE[1]: The classification of annotated substances was carried out according to the Classyfire database. NOTE[2]: The nomenclature provided by the database records was maintained in the breakdown of compounds.

Table S2. Apparent viscosity (30 s) and area of hysteresis of the creams as a function of storage time at room temperature.

Time (day)	CBlank		CPropolis	
	Viscosity (Pa.s)	Area of hysteresis	Viscosity (Pa.s)	Area of hysteresis
0	2.9 ± 0.2 *	14,959 ± 1171 *	1.7 ± 0.5	11,783 ± 309
15	3.0 ± 0.1	15,653 ± 909	2.9 ± 0.2	14,296 ± 3319
30	3.2 ± 0.5	12,166 ± 452	3.2 ± 0.1	11,689 ± 1586
45	3.7 ± 1.1	11,220 ± 1214	2.3 ± 0.3	13,595 ± 2301
60	3.7 ± 0.2	12,400 ± 2586	3.5 ± 0.7 **	14,379 ± 3152

Values expressed as mean ± SD. *t*-test for the same times of different samples and ANOVA, with Tukey's post hoc test for all times of the same sample ($p < 0.01$) ($n = 3$). * Statistical difference between the T0 of the two samples. ** Statistical difference of the same sample in relation to the T0.

Table S3. Confounding variables taken into account in the distribution of groups.

Population	Control Group ($n = 8$)	Frequency (%)	Intervention Group ($n = 8$)	Frequency (%)	*p*-value
MTX dose increase	1	12.5	0	0	
Introduction of oral corticosteroids	0	0	1	12.5	
Introduction of oral antibiotics and topical corticosteroids	1	12.5	0	0	0.608 *
MTX withdrawal	0	0	1	12.5	
Cyclosporine dose decrease	0	0	1	12.5	
Oral corticosteroids withdrawal	0	0	1	12.5	

MTX= methotrexate. * Probability of significance (*p*-value) calculated by Fisher's exact test.

Table S4. Allergic comorbidities presented by patients included in the study.

Comorbidities	Control Group ($n = 8$)	Frequency (%)	Intervention Group ($n = 8$)	Frequency (%)
Rhinit	3	37.5	3	37.5
Asthma	2	25	0	0
Asthma and Rhinit	0	0	3	37.5

Figure S3. Sociodemographic and clinical data.

ATOPIC DERMATITIS RESEARCH – FCFRP/USP

SOCIODEMOGRAPHIC AND CLINICAL DATA

Name: ______________________________ Telephone: ______________________
Registration N°: ______________ Adress: ______________________
Date of 1st interview: ___/___/___ Date of 2nd interview: ___/___/___ Date of 3rd interview: ___/___/___

SOCIODEMOGRAPHIC DATA

1. Gender: ☐ M ☐ F 2. Pregnant or Lactating? ☐ No ☐ Yes 3. Age: ________

4. Birth: ___/___/___ 5. Marital Status: ☐ Single ☐ Married ☐ Widower ☐ Divorced

Others: ______________

6. Education Level: ☐ Illiterate ☐ Elementary school ☐ Incomplete elementary school ☐ Midlle school ☐ Higher education ☐ Postgraduate

7. Profession: ______________

8. Profissional situation: ☐ Employed ☐ Unemployed ☐ Student ☐ Retiree

9. Companion: ☐ No ☐ Yes
Who? ________________________ Telephone: ______________

CLINICAL DATA

10. First medical diagnosis of atopic dermatitis: ______________________

11. Number of dermatologist appointments per year: __________

12. Do you have contact dermatitis? ☐ No ☐ Yes To which material? ______________________

12.1 Avoid contact? ☐ No ☐ Yes To which material? ______________________

13. Perform environmental prophylaxis? ☐ No ☐ Yes

14. Do you have dogs? ☐ No ☐ Yes

15. Do you have cats? ☐ No ☐ Yes

16. Do you use sofa cover? ☐ No ☐ Yes 17. Do you have rugs at home? ☐ No ☐ Yes

18. Are you allergic to any drug? ☐ No ☐ Yes Which one? ______________________

19. Are you allergic to propolis (substance present in bee honey)? ☐ No ☐ Yes ☐ I do not know

20. Are you allergic to cocoa butter and/or cocoa? ☐ No ☐ Yes ☐ I do not know

21. The use of cosmetic was recommended by the doctor?(moisturizing cream): ☐ No ☐ Yes Which?______

22. Do you use cosmetics as a complement to the treatment of atopic dermatitis? ☐ No ☐ Yes Which?____

23. What is the bath time?___________ 23.1 Do you use bath sponge? ☐ No ☐ Yes

23.2 Do you use warm water? ☐ No ☐ Yes

24. Have you ever had any previous surgery? ☐ No ☐ I do not know ☐ Yes, Which?______

☐ Tonsillectomy ☐ Adenectomy Others:____________________

25. Do you have allergic diseases? ☐ No ☐ I do not know ☐ Yes, Which?___________

☐ Allergic Rhinits ☐ Asthma ☐ Allergic conjunctivitis Others:______________________

26. Do you have other diagnosed diseases? ☐ No ☐ Yes, Which?_________________

☐ Liver Diseases ☐ Depression ☐ Neurological Diseases

☐ Anxiety ☐ Kidney Diseases ☐ Cardiovascular Diseases

☐ Metabolic Diseases ☐ Osteo-articular Diseases Others:______________________

27. Is there another family member with atopic dermatitis? ☐ No ☐ Yes Who?_______________

28. What is the period of the year/weather when the atopic dermatitis gets worse? ☐ Summer ☐ Winter ☐ Rain ☐ Indifferent Others________

29. How do you consider your illness? ☐ Mild ☐ Moderate ☐ Severe

30. Do you understand how to use the cream? ☐ Yes ☐ No

DRUG THERAPY

Drug (Oral)	How many times/days and what time	Continuous use (yes or no)	Treatment time
**Drug (Topical)**	**How many times/days and what time**	**Continuous use (yes or no)**	**Treatment time**

Drug (Injectable)	How many times/days and what time	Continuous use (yes or no)	Treatment time
**Drug (Sublingual)**	**How many times/days and what time**	**Continuous use (yes or no)**	**Treatment time**
**Drug (Nasal)**	**How many times/days and what time**	**Continuous use (yes or no)**	**Treatment time**

INJURIES REGIONS

31. Presence of injuries 0 month:

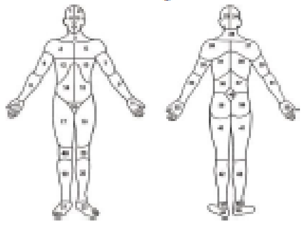

Presence of injuries 2 month:

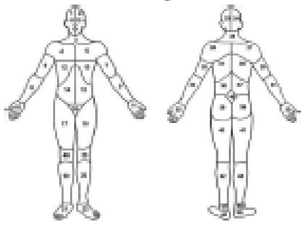

HEALTH BEHAVIORS

32. Do you consume alcoholic drink? ☐ No ☐ Yes Quantity per day:________________

33. Do you smoke? ☐ No ☐ Ex Smoker ☐ Yes Quantity per day:_____________

34. Do you exercise? ☐ No ☐ Yes Frequency:_______________________

DATA ON FORMULATION ADHESION – AFTER 1 MONTH OF TREATMENT

35. Have you ever stopped using the cream? ☐ No ☐ Yes How many days:___________Why?__________

36. Do the cream cause any discomfort? ☐ No ☐ Yes Which:_____________________________

37. Regarding the cream, what are the complaints below:

☐ Inefficient treatment ☐ Treatment interferes with daily activities ☐ Fear of side effects

☐ Instructions are unclear ☐ Cream has unpleasant smell ☐ Cream caused itching

☐ Cream leaves stains on clothes or surface

☐ Cream changed appearance ☐ Cream left the skin dry

☐ Cream caused burning ☐ Cream makes the skin oily

☐ Crem is difficult to spread ☐ Cream is hard to get out of the bottle

Others:____

DATA ON FORMULATION ADHESION – AFTER 2 MONTH OF TREATMENT

38. Have you ever stopped using the cream? ☐ No ☐ Yes How many days:__________ Why?__________

39. Do the cream cause any discomfort? ☐ No ☐ Yes Which:____________________________

40. Did you use any other moisturizer during the study period? ☐ No ☐ Yes Why?_____________

41. Is there any cream left in the bottle? ☐ No ☐ Yes 41.1 How many bottles?_______________

42. Regarding the cream, what are the complaints below:

☐ Inefficient treatment ☐ Treatment interferes with daily activities ☐ Fear of side effects

☐ Instructions are unclear ☐ Cream has unpleasant smell ☐ Cream caused itching

☐ Cream leaves stains on clothes or surface

☐ Cream changed appearance ☐ Cream left the skin dry

☐ Cream caused burning ☐ Cream makes the skin oily

☐ Crem is difficult to spread ☐ Cream is hard to get out of the bottle

Others:____

LABORATORY EXAMS

43. IgE Values:

44. Prick test: ☐ Accomplished ☐ Unaccomplished

To which elements? ____

45. Patch test: ☐ Accomplished ☐ Unaccomplished

To which elements and number of crosses? ____
